# Myelosuppression in Patients Treated with the Telomerase Inhibitor Imetelstat Is Not Mediated through Activation of Toll-Like Receptors

**DOI:** 10.3390/ijms21186550

**Published:** 2020-09-08

**Authors:** Gabriela M. Baerlocher, Joshua Rusbuldt, Jacqueline Bussolari, Fei Huang

**Affiliations:** 1 Department of Hematology and Central Hematology Laboratory, Inselspital, Bern University Hospital, University of Bern, 3010 Bern, Switzerland; 2 Laboratory for Hematopoiesis and Molecular Genetics, Department of BioMedical Research (DBMR), University of Bern, 3010 Bern, Switzerland; 3 Janssen Research & Development, LLC, Spring House, PA 19477, USA; jrusbul3@its.jnj.com (J.R.); JBUSSOLA@ITS.JNJ.COM (J.B.); 4 Geron Corporation, Parsippany, NJ 07054, USA; fhuang@Geron.com

**Keywords:** imetelstat, thrombocytopenia, Toll-like receptor, telomerase inhibitor, oligonucleotide

## Abstract

Imetelstat sodium (GRN163L; hereafter, imetelstat) is a first-in-class telomerase inhibitor that has demonstrated activity in patients with myeloproliferative neoplasms (MPNs). Treatment with imetelstat has been associated with thrombocytopenia and other hematologic adverse effects that were manageable and reversible. Toll-like receptors (TLRs) are proteins that recognize pathogen-associated molecular patterns and stimulate innate immune and pro-apoptotic responses. Because imetelstat is an oligonucleotide, and some oligonucleotides can activate TLRs, we conducted an in vitro study to rule out the possibility of imetelstat-associated thrombocytopenia by off-target effects through activation of TLRs. We used HEK293 cell lines stably co-expressing a human TLR gene and an NFκB-inducible reporter to investigate whether imetelstat can activate TLR signaling. We treated the cells with imetelstat or control oligonucleotides for 20 h, and used absorbance of the culture media to calculate the reporter activity. Treatment with imetelstat within or beyond the clinically relevant concentrations had no stimulatory effect on TLR2, TLR3, TLR4, TLR5, TLR7, or TLR9. This result was not surprising since the structure of imetelstat does not meet the reported minimal structural requirements for TLR9 activation. Furthermore, imetelstat treatment of the MPN cell line HEL did not impact the expression of TLR signaling pathway target genes that are commonly induced by activation of different TLRs, whereas it significantly reduced its target gene *hTERT*, human telomerase reverse transcriptase, in a dose- and time-dependent manner. Hence, cytopenias, especially thrombocytopenia observed in some patients treated with imetelstat, are not mediated by off-target interactions with TLRs.

## 1. Background

Imetelstat has demonstrated activity in patients with myeloproliferative neoplasms, including essential thrombocythemia [1]; primary, post-essential thrombocythemia, or post-polycythemia vera myelofibrosis [2,3]; and lower-risk myelodysplastic syndromes [4,5]. Imetelstat is a covalently lipidated 13-mer oligonucleotide that is complementary to the RNA template region of the telomerase enzyme [6], and acts as a potent direct competitive inhibitor of telomerase reverse transcriptase enzyme activity (half-maximal inhibitory concentration in cell-free assays = 0.5–10.0 nM) [6,7,8]. By binding to the RNA component, imetelstat inhibits telomerase activity, mediating downstream reduction in telomere length [6,7,8], which leads to its effects in malignant cells. Indeed, telomerase inhibition by imetelstat has been reported across a number of cancer cell lines and primary human cells *in vitro*, as well as in human cells in vivo [6,7,9].

Toll-like receptors (TLRs) are proteins with domains that recognize pathogen-associated molecular patterns and stimulate downstream signaling to trigger innate immune and pro-apoptotic responses [10]. There are at least 10 TLRs in humans, and they are specific for different molecular patterns [10]. For example, lipopeptides, double-stranded RNA, lipopolysaccharide, and flagellin activate TLR2, TLR3, TLR4, and TLR5, respectively; TLR9 is activated by deoxycytidylate-phosphate-deoxyguanylate DNA [10]. In addition to DNA from pathogens, TLR9 can be activated by synthetic, single-stranded DNA with CpG (nonmethylated deoxycytidyldeoxyguanosine) dinucleotide motifs that are characteristic of bacterial and viral genomes [11,12]. Upon recognition of specific ligands, TLRs initiate intracellular signaling cascades that induce a broad gene expression program to regulate the defense against pathogens and stimulate adaptive immune responses [13]. The different TLRs ensure an effective response against a wide variety of microbial pathogens by inducing a common set of gene products with general antimicrobial and immunomodulatory properties [14].

Treatment with imetelstat has been associated with thrombocytopenia, neutropenia, and reduced hemoglobin levels [1,2]. In a phase II study of imetelstat in patients with essential thrombocythemia (*n* = 18) who had not responded to or had unacceptable side effects with prior treatment, 83% reported anemia (*n* = 15; grade ≥3 anemia was reported in 17% (*n* = 3)), 83% reported neutropenia (*n* = 15; grade ≥3 was reported in 56% (*n* = 10)), and 50% reported thrombocytopenia of any grade (*n* = 9; none were grade ≥ 3) [1]. Thrombocytopenia resolved with imetelstat discontinuation; patients with longer treatment intervals had more gradual increases in platelet counts compared with those who received more frequent dosing [1]. In the pilot study of imetelstat in patients with high-risk or intermediate-2-risk myelofibrosis (*n* = 33), 45% (*n* = 15) reported thrombocytopenia (all events were grade ≥ 3), 39% (*n* = 13) reported anemia (grade 3 in 30% (*n* = 10)), and 27% (*n* = 9) reported neutropenia (all events were grade ≥ 3); all of these events were considered to be related to treatment [2].

Given that imetelstat is an oligonucleotide, and some oligonucleotides can activate TLRs, it has been speculated that the thrombocytopenia observed in patients with myeloproliferative neoplasms treated with imetelstat may be mediated by off-target effects through the activation of TLRs such as TLR9 [15]. It has been further speculated that such off-target effects might be the primary mechanism of action of imetelstat in patients with myeloproliferative neoplasms [15]. In murine models, thrombocytopenia can be induced by TLR activation, specifically via TLR4 [16]. However, no evidence of direct TLR-induced thrombocytopenia in humans has been reported.

## 2. Results and Discussion

We investigated whether imetelstat activates TLRs and, by extension, whether the thrombocytopenia observed in some patients treated with imetelstat may be due to the off-target stimulation of these receptors. Using an NFκB-inducible secreted embryonic alkaline phosphatase (SEAP) reporter assay for TLR activity we assayed imetelstat at clinically relevant or higher concentrations (10 µM, 30 µM, and 60 µM), as well as control oligonucleotides (mismatched or non-complementary sequences) at the same concentrations (Table 1). We also investigated positive control ligands specific to each TLR.

**Figure 1:**
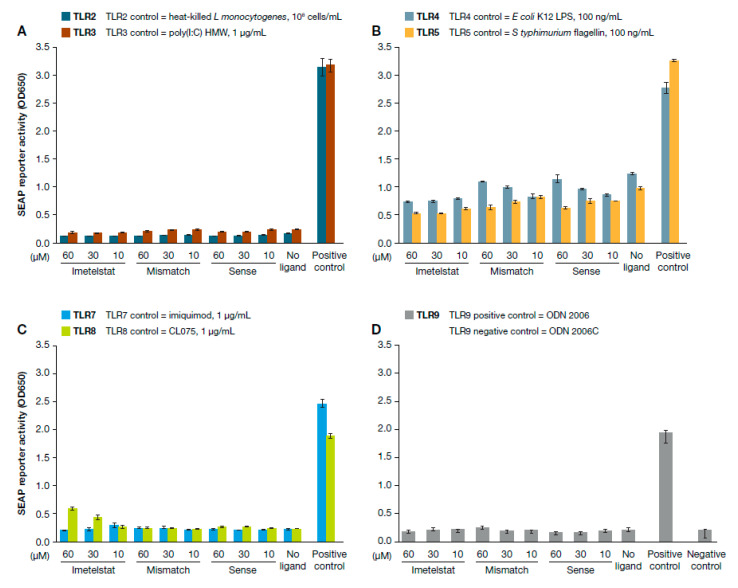
Effects of imetelstat on TLR activity as measured by NFκB-inducible secreted embryonic alkaline phosphatase (SEAP) reporter assay. HEK-Blue TLR cells were incubated with different positive control ligands or three concentrations of imetelstat. After 20 h incubation, the cells were measured for absorbance at 650 nm for SEAP activity. Each TLR experiment was performed twice with three replicates per experiment. Mean values with standard derivations are shown. Effects of imetelstat on (**A**) TLR2 and TLR3 receptors; (**B**) TLR4 and TLR5 receptors; (**C**) TLR7 and TLR8 receptors; and (**D**) TLR9 receptors.

Treatment with various concentrations of imetelstat even at concentrations higher than clinically relevant showed that SEAP reporter activity resulted in no significant difference compared to untreated cell controls, indicating no stimulatory effect on TLR2, TLR3, TLR4, TLR5, TLR7, or TLR9; neither mismatch nor sense oligonucleotides stimulated the TLRs in this study (Figure 1). For TLR8, treatment with imetelstat at the clinically relevant concentration of 10 µM did not increase the reporter activity compared to that in the untreated control (1.1-fold, *p* = 0.26), although a dose-dependent increase of SEAP reporter activity was observed. Compared to the untreated control, 30 µM and 60 µM of imetelstat had 1.9-fold (*p* = 0.019) and 2.6-fold (*p* = 0.009) higher reporter activity, respectively; however, both concentrations were higher than the maximal concentration of imetelstat achieved clinically with the optimal dose. In contrast, compared to the positive control treatment, different concentrations of imetelstat treatment yielded significantly lower SEAP reporter activity (Figure 1C). Imetelstat at 10 µM, 30 µM, and 60 µM had 7.3-fold (*p* = 2 × 10^−9^), 4.3-fold (*p* = 1 × 10^−6^), and 3.2-fold (*p* = 2.8 × 10^−5^) less reporter activity than the positive control, respectively. It is not clear why imetelstat at concentrations higher than clinical exposure showed stimulation of TLR8. It has been reported that TLR8 can recognize G-rich single-stranded RNA oligonucleotides from HIV-1 [17], but imetelstat does not share the sequence homology.

**Figure 2:**
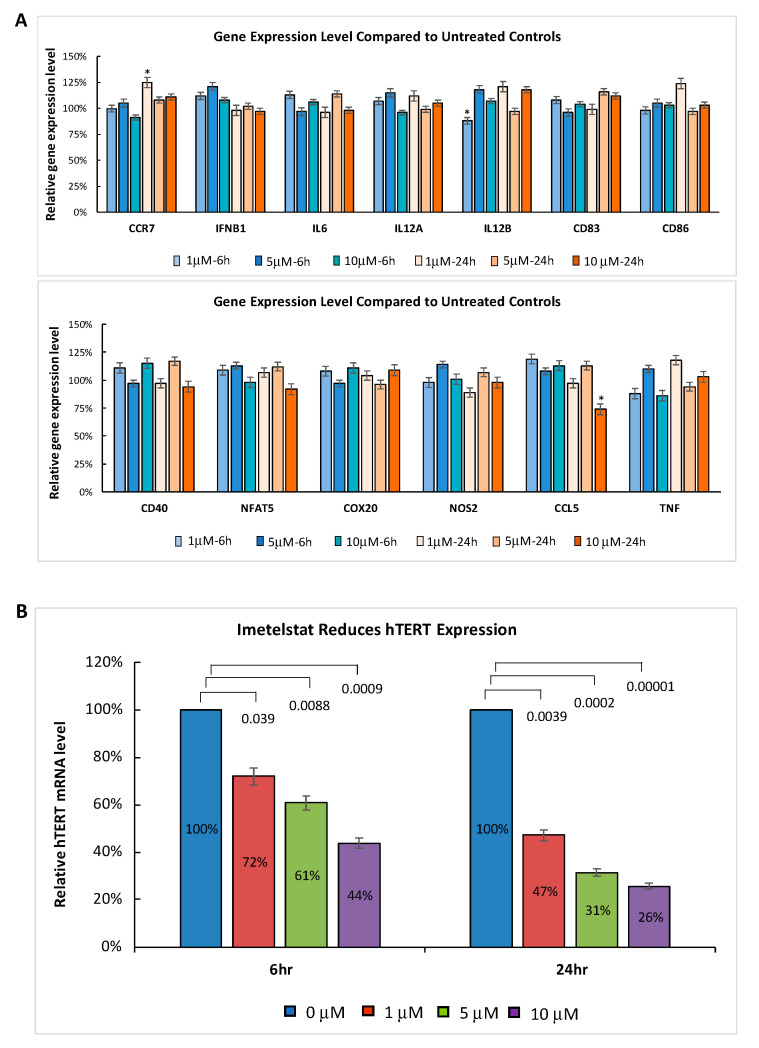
MPN HEL cells were treated with 0, 1 µM, 5 µM, or 10 µM imetelstat for 6 and 24 h in triplicate. RNA was isolated and gene expression profiling was performed using the Affymetrix GeneChip. The mean percentage of gene expression induced by imetelstat treatment at different concentrations was compared to the corresponding untreated control at given time points. Error bars are standard deviations. (**A**) Imetelstat did not significantly induce the expression of the TLR signaling pathway target genes (* *p* < 0.05). (**B**) Dose-dependent reduction of *hTERT* RNA expression level by imetelstat treatment. The *p*-values are indicated.

To confirm that imetelstat does not activate TLRs, we further explored whether imetelstat had any impact on the expression of TLR signaling pathway target genes. The myeloproliferative neoplasm (MPN) cell line HEL was treated with different clinically relevant concentrations of imetelstat for 6 and 24 h. Gene expression profiling was performed using the Affymetrix GeneChip™. We evaluated the expression of a set of genes (*CCR7*, *IFNB1*, *IL6*, *IL12A*, *IL12B*, *CD83*, *CD86*, *CD40*, *NFAT5*, *COX20*, *NOS2*, *CCL5,* and *TNF*) that are reported to be commonly induced by the activation of different TLRs upon the recognition of specific ligands [13,14]. Imetelstat at clinically relevant concentrations did not significantly change the expression of these TLR signaling pathway target genes (Figure 2A). Although there were three genes (*CCR7*, *CCL5*, and *IL12B*) with *p*-value < 0.05, none of them showed dose-dependent changes. The lack of induction in TLR signaling pathway target genes further supports that imetelstat did not activate TLRs. In contrast, imetelstat induced significant dose- and time-dependent decreases in the expression of its target gene *hTERT* (human telomerase reverse transcriptase), indicating that the mechanism of action is via on-target activity (Figure 2B).

It was not surprising that imetelstat did not activate TLRs in the reporter in vitro assays or significantly change the expression of the TLR signaling pathway target genes since the 13-mer structure of imetelstat is shorter than the minimal sequence required for TLR9 activation (Table 2) [12]. The minimal structural requirements for the oligonucleotide activation of human TLR9 are two CpG islands separated by 6–10 nucleotides, where the first CpG motif is preceded by 5′-thymidine and followed by an elongated (≥10 nucleotides) poly-thymidine tail at the 3′ end [12]. Oligonucleotides shorter than 21 nucleotides are less likely to activate TLR9 [12]. Additionally, imetelstat lacks the CpG motifs needed to activate TLR9.

Megakaryocytes and platelets express various TLRs. Although the function of TLRs in megakaryocytes is not completely understood, TLRs on platelets have been more extensively studied, particularly for TLR2 and TLR4. Both allow platelets to recognize pathogens and augment platelet activation in order to regulate platelet immunity and function [18]. Thrombocytopenia is a frequent complication of viral infections. Depending on the nature of the infecting viruses, several mechanisms including immunologic platelet destruction, increased platelet clearance, inappropriate platelet activation and consumption, as well as impaired megakaryopoiesis are involved [19]. TLR3, TLR4, TLR7, TLR8, and TLR9 recognize viral components and induce antiviral responses, but their role in viral-infection-associated thrombocytopenia is not completely understood. A recent study showed that monocytes differentiated into inflammatory hemophagocytes via TLR7 and TLR9 signaling led to anemia and thrombocytopenia [20]. Our results revealed that imetelstat did not activate TLR7 and TLR9 signaling; therefore, cytopenias observed in patients treated with imetelstat are unlikely due to activation of TLRs. Although imetelstat at higher concentrations stimulated TLR8 in the reporter assay, to our knowledge, there is no published report directly linking TLR8 with thrombocytopenia.

As we are unaware of any direct evidence from the literature that the activation of TLRs can cause thrombocytopenia in humans, we maintain that the mechanism underlying cytopenia associated with imetelstat therapy must be mediated by other means. Since imetelstat has been associated with certain hematologic adverse events (i.e., neutropenia, reduced hemoglobin levels) [1,2], it has been proposed that treatment may cause on-target effects on hematologic stem and progenitor cells. As megakaryocytic differentiation requires the upregulation of telomerase activity [21], thrombocytopenia in patients treated with imetelstat is likely a downstream effect of on-target telomerase inhibition. Indeed, work performed by another group has indicated that the thrombocytopenic effects of imetelstat may be mediated by changes in the megakaryocytic progenitor cells that produce platelets [22]. Specifically, telomerase inhibition by imetelstat prevents normal maturation of megakaryocyte precursor cells [22]; the specific stage or stages of megakaryocyte maturation affected by imetelstat inhibition are not yet clearly defined. Blockade of maturation can then create an accumulation of immature megakaryocyte cells, thus reducing the number of available mature platelets (Graphical Abstract). The effects of imetelstat are also different in neoplastic cells compared with normal cells. Imetelstat treatment ex vivo inhibits megakaryocyte colony-forming units (CFU-MKs) in samples from patients with essential thrombocythemia but not CFU-MKs in samples from healthy individuals [23]. These results were supported by the inhibition of telomerase and clonal proliferation of megakaryocyte in samples from patients with essential thrombocythemia in a phase II study of imetelstat [1]. Additionally, it has been demonstrated that imetelstat is capable of depleting myelofibrosis stem and progenitor cells [24]. Furthermore, telomerase activity is upregulated during erythroid differentiation, reaches levels comparable to those in tumor cell lines in erythroid precursor cells, and correlates exclusively with erythroid proliferative potential [25]. Telomerase is highly regulated during the development of hematopoietic cells to have an impact on the production of myeloid lineage cells [26]. Telomerase is highly activated in normal hematopoietic stem cells to maintain self-renewal capacity, and transiently expressed in myeloid progenitor cells, but downregulated in mature myeloid cells [26], while telomerase is constitutively activated in malignant hematopoietic cells and stem cells [27]. To better understand the mechanisms underlying imetelstat’s beneficial effects in patients with myeloid malignancies and the therapy-related cytopenias [1,2,3,4,5], further studies on the impact of imetelstat on normal and dysplastic erythro- and myelopoiesis are warranted.

In conclusion, because imetelstat did not activate TLRs and had no impact on the expression of TLR signaling pathway target genes, the cytopenias, including anemia, neutropenia, and thrombocytopenia, observed in some patients treated with imetelstat are not mediated by off-target interactions with TLRs. These findings are supported by the structural differences between imetelstat and the minimal requirements to activate TLR9 [12], and by earlier results from our group and others demonstrating the effects of imetelstat on megakaryocytes through mechanisms other than TLRs [1,22,23]. We instead hypothesize that cytopenias and especially the thrombocytopenia associated with imetelstat treatment results from on-target effects on the stem and progenitor cell pool.

## 3. Materials and Methods

Seven HEK293 cell lines, each stably co-expressing a human TLR gene (encoding hTLR2, hTLR3, hTLR4, hTLR5, hTLR7, hTLR8, or hTLR9) and an NFκB-inducible SEAP reporter gene (HEK-Blue™ TLR cells) and TLR ligands as controls (all from InvivoGen, San Diego, CA, USA) were used to test whether experimental agents (see Table 1) activated TLR signaling. Cells were grown per the manufacturer’s recommendation; briefly, each TLR-expressing line was cultured in DMEM with 10% fetal bovine serum (FBS) (both from Gibco, Dublin, Ireland) containing 50 µg/mL streptomycin and 100 µg/mL Normocin as selection markers (included with InvivoGen kit) until approximately 70% confluent. At the time of assay, cells were gently removed via scraping and reseeded at 50,000 cells per well into opaque 96-well culture plates (Perkin Elmer, Waltham, MA, USA) and then treated with 10 µM, 30 µM, or 60 µM imetelstat, control oligonucleotides (mis-matched and sense oligonucleotides), or corresponding positive or negative control ligands for each TLR (see Table 1) at one-tenth culture volume for 20 h. All test compounds and controls, as well as dilutes, were prepared in sterile water (Invitrogen, Waltham, MA, USA). The absorbance of the cell culture in detection media at OD650 nm wavelength was determined on a SpectraMax M5 spectrophotometer (Molecular Devices, San Jose, CA, USA) and used to calculate SEAP reporter activity based on absorbance compared to untreated (media only) wells. Each TLR experiment was performed twice, with three replicates per experiment. A *t*-test was performed to compare each treatment group to its corresponding untreated control and positive control for each TLR study.

Human erythroleukemia cell line HEL 92.1.7 (ATCC^®^ TIB-180™) was cultured in RPMI-1640 with 10% FBS. Cells were grown to 70% confluence, then treated with 1 µM, 5 µM, or 10 µM imetelstat for 6 or 24 h in triplicate. Untreated cells served as controls. Total RNA was purified using the AllPrep Mini Kit (Qiagen, Germantown, MD, USA) on the QiaCube sample processor, per kit instructions. Transcriptome profiling was performed using the Affymetrix GeneChip™ Human Gene 2.0 ST Array (Affymetrix, Inc. Santa Clara, CA, USA), per the manufacturer’s recommendation. The gene expression profiles were compared between imetelstat-treated cells and untreated controls.

## Figures and Tables

**Table 1:** Ligands for the Toll-like receptor (TLR) activation experiment.

Experimental Agents ^a^		
Imetelstat	5′-R-TAGGGTTAGACAA-NH_2_-3′	
Mismatch oligonucleotide (with 4 nucleotides mismatched to imetelstat)	5′-R-TAGGTGTAAGCAA-NH_2_-3′	
Sense oligonucleotide (with reverse sequence of imetelstat)	5′-AACAGATTGGGAT-R-3′	
**Positive Control Agents**		
**Positive Control Ligand**	**Receptor**	**Ligand Concentration**
Heat-killed *Listeria monocytogenes*	hTLR2	10^8^ cells/mL
Poly(I:C) HMW	hTLR3	1 μg/mL
*Escherichia coli* K12 lipopolysaccharide	hTLR4	100 ng/mL
*Salmonella typhimurium* flagellin	hTLR5	100 ng/mL
Imiquimod	hTLR7	1 μg/mL
CL075	hTLR8	1 μg/mL
CpG ODN 2006	hTLR9	100 ng/mL

Abbreviations: CpG, cytosine triphosphate deoxynucleotide followed by a guanine triphosphate deoxynucleotide with a phosphodiester link between; HMW, high molecular weight; ODN, oligodeoxynucleotide; poly (I:C), polyinosinic-polycytidylic acid. ^a^ R in oligonucleotide sequences refers to the covalently bound lipophilic (palmitoyl) group.

**Table 2:** Features required for TLR9 activation compared to imetelstat sequence [12].

Requirement	Imetelstat	Conclusion
At least two CpG islands separated by 6 to 10 nucleotides, e.g., 5′-TCGTTTTTTTCGTTTTTTTTTTTT-3′	13-mer oligonucleotide: 5′-TAGGGTTAGACAA-NH_2_-3′	Criteria not met
First CpG island is preceded by a 5′ thymidine, e.g., 5′-TCGTTTTTTTCGTTTTTTTTTTTT-3′		Criteria not met
Poly-thymidine tail of ≥10 nucleotides at the 3′ end, e.g., 5′-TCGTTTTTTTCGTTTTTTTTTTTT-3′		Criteria not met
Overall length ≥21 nucleotides		Criteria not met
